# Role of efflux pumps, their inhibitors, and regulators in colistin resistance

**DOI:** 10.3389/fmicb.2023.1207441

**Published:** 2023-08-04

**Authors:** Yinhuan Ding, Jingchen Hao, Weijia Xiao, Caihong Ye, Xue Xiao, Chunxia Jian, Min Tang, Guangrong Li, Jinbo Liu, Zhangrui Zeng

**Affiliations:** Department of Laboratory Medicine, the Affiliated Hospital of Southwest Medical University, Luzhou, China

**Keywords:** efflux pump, colistin resistance, lipopolysaccharide, efflux pump inhibitors, collateral susceptibility, heteroresistance

## Abstract

Colistin is highly promising against multidrug-resistant and extensively drug-resistant bacteria clinically. Bacteria are resistant to colistin mainly through *mcr* and chromosome-mediated lipopolysaccharide (LPS) synthesis-related locus variation. However, the current understanding cannot fully explain the resistance mechanism in *mcr*-negative colistin-resistant strains. Significantly, the contribution of efflux pumps to colistin resistance remains to be clarified. This review aims to discuss the contribution of efflux pumps and their related transcriptional regulators to colistin resistance in various bacteria and the reversal effect of efflux pump inhibitors on colistin resistance. Previous studies suggested a complex regulatory relationship between the efflux pumps and their transcriptional regulators and LPS synthesis, transport, and modification. Carbonyl cyanide 3-chlorophenylhydrazone (CCCP), 1-(1-naphthylmethyl)-piperazine (NMP), and Phe-Arg-β-naphthylamide (PAβN) all achieved the reversal of colistin resistance, highlighting the role of efflux pumps in colistin resistance and their potential for adjuvant development. The contribution of the efflux pumps to colistin resistance might also be related to specific genetic backgrounds. They can participate in colistin tolerance and heterogeneous resistance to affect the treatment efficacy of colistin. These findings help understand the development of resistance in *mcr*-negative colistin-resistant strains.

## Introduction

The emergence and widespread occurrence of carbapenem-resistant gram-negative bacteria (CRGB: *Enterobacteriaceae*, *Pseudomonas* spp., *Acinetobacter* spp., etc.) poses a severe public health risk, with some metallo-β-lactamases-producing individuals also revealing inherent resistance to ceftazidime/avibactam. These CRGB acquire multiple antimicrobial resistance determinants under the action of mobile elements, promoting their resistance to tetracycline, quinolone, aminoglycosides, and other antibiotics. Additionally, energy-dependent efflux pumps, the classical pathway of bacterial resistance, play an essential role in multidrug resistance in bacteria. The efflux pumps can be divided into resistance-nodulation-cell division (RND), major facilitator superfamily (MFS), multidrug and toxic compound extrusion (MATE), small multidrug resistance (SMR), and ATP-binding cassette (ABC) super-families based on the differences in efflux transport proteins (Li and Nikaido, 2009). These transporters have a wide range of efflux substrates covering antibiotics for common clinical anti-infective treatments (β-lactams, tetracyclines, chloramphenicol, fluoroquinolones, aminoglycosides, sulfonamides, etc.), besides being involved in the elimination of bacterial intracellular metabolites. In the clinical infection setting, these efflux pumps also eliminate a wide range of chemical disinfectants favoring the long-term colonization of an abiotic surface by pathogens. These efflux pumps also respond quickly to enhance their viability when exposed to harsh environments (Fernández and Hancock, 2012). In particular, efflux pump transcription regulators are essential in regulating oxidative stress, physiological metabolism, and fitness (Holden and Webber, 2020). These results suggest that the efflux pumps are also an efficient means for bacteria to resist external environmental stress. Understanding their operation and development can help understand the evolutionary process of bacteria.

As carbapenems failed to treat CRGB, colistin (polymyxin B and E) was reintroduced to treat some refractory gram-negative infections. Colistin, as a cationic polypeptide compound, mainly acts on the outer membrane of bacteria carrying a negative charge. Colistin can destroy the stability of the outer membrane by displacing Ca^2+^ and Mg^2+^ ions, thus playing a bactericidal role (Falagas and Kasiakou, 2005). However, the lipid modification mediated by mutation and the inactivation of the two-component regulatory systems (TCS: PmrAB and PhoPQ) and *mgrB* are common causes of reduced susceptibility to colistin in bacteria. Plasmid-mediated horizontal transfer of *mcr* and its variants in bacteria promotes the rapid emergence of colistin resistance. In contrast, the role of efflux pumps in colistin resistance has received less attention. Previous studies have shown that efflux pump inhibitors (EPIs) CCCP and NMPs reverse colistin resistance, suggesting the role of efflux pumps in colistin resistance. Despite colistin’s much larger size than other common antibiotics and chemicals, the efflux of colistin compounds may be used as efflux substrates have been observed in many bacteria (Warner and Levy, 2010; Sundaramoorthy et al., 2019a). LPS modification and overexpression of efflux pumps and their regulators are also thought to be important components of the colistin resistance mechanisms in mcr-negative *K. pneumoniae* (Naha et al., 2022). In January 2023, we conducted a search on PubMed and Web of Science databases using keywords such as “colistin,” “efflux pump,” and “cationic antimicrobial peptide.” We screened over 100 publications summarized in this review, aiming to explore the role of the efflux pump in colistin resistance and provide a basis for elucidating the resistance mechanisms of mcr-negative colistin-resistant strains. The flow chart depicting the search and selection process is provided in Supplementary Figure S1.

## General and novel colistin resistance mechanisms

The absence of the outer membrane limits the use of colistin against gram-positive bacteria. The colistin resistance mechanisms in gram-negative bacteria have been summarized in many previous reviews (Jeannot et al., 2017; Gogry et al., 2021). In general, the colistin resistance mechanism mainly includes two steps. First, point mutations in the TCS PmrAB, PhoPQ, CrrAB, and other lipid A modification coding genes located in chromosome loci produce more positively charged phosphoethanolamine to be added to the outer membrane lipids, resulting in weakened binding to colistin. Mutational inactivation and truncation of the insertion sequence of the PhoQ kinase inhibitor *mgrB* may also be effectively involved in this process. Second, phosphoethanolamine transferase encoded by *mcr* can also mediate the modification of the outer membrane (lipid A). More than 100 variants of *mcr* have been identified since it was first reported in 2015 (Liu et al., 2016).^1^ The *mcr* variants have completed horizontal transfer among different species with the help of mobile elements, posing a severe threat to public health. Compensatory mutations can effectively alleviate the fitness costs of *mcr* and promote the continued existence of plasmids carrying *mcr* (Yang et al., 2020). A recent study on *A. hydrophila* suggests that MlaA, the outer membrane lipoprotein-encoding gene, may be associated with high levels of colistin resistance (Liu et al., 2021). A recent study found that the RpoE stress system mediated colistin resistance in *E. coli* without disturbing the lipid A profile (Zeng et al., 2023). The discovery of colistin-degrading proteases also reveals the diversity of colistin resistance mechanisms (Lee et al., 2022).

## Impacts of EPIs on colistin resistance

Although colistin is one of the few treatments available for multi-drug resistant pathogens, it does not seem effective in reducing patient mortality (Kelesidis and Falagas, 2015; Lee et al., 2020). Therefore, effective alternative treatment measures need to be developed. Current reviews of colistin resistance mechanisms have described less about the contribution of efflux pumps. Ni et al. (2016) successfully restored colistin susceptibility in colistin-resistant *A. baumannii*, *P. aeruginosa*, *K. pneumoniae*, and *S. maltophilia* using the efflux pump inhibitor CCCP (Table 1). Subsequent studies demonstrated that CCCP reversed colistin resistance in gram-negative bacteria that produced or did not produce *mcr* (Osei Sekyere and Amoako, 2017; Baron and Rolain, 2018). In contrast, other common EPIs (reserpine, verapamil, PAβN, and NMP) have more pronounced effects on the MICs of non-colistin-resistant strains (Table 1) (Ni et al., 2016; Osei Sekyere and Amoako, 2017). DNP, another proton-carrier inhibitor similar to CCCP, has apparent effects on colistin-resistant *A. baumannii* (Park and Ko, 2015). These results suggested that the differences in EPIs might be the main reason influencing the restoration of colistin susceptibility. The possible explanation is that CCCP-mediated electrochemical gradient depolarization can restore the negative charge of the outer membrane and lead to increased susceptibility to bacteria (Ni et al., 2016). Similarly, the MarR inhibitor salicylic acid can affect the negative cell surface charge of colistin-resistant *E. coli* and restore colistin susceptibility, but this effect is not apparent in colistin-sensitive *E. coli* (Sundaramoorthy et al., 2019b). Meanwhile, CCCP can reduce the metabolic activity of *A. baumannii* and increase its susceptibility to colistin (Park and Ko, 2015). A study in *P. aeruginosa* also showed that CCCP affected energy metabolism and thus decreased the colistin tolerance of *P. aeruginosa* biofilms (Pamp et al., 2008). In contrast, a previous study showed that CCCP contributed to the increase in polymyxin B resistance in wild-type and *phop* mutant in survival assays, which was mainly explained by the reduction of the proton gradient in the bacterial inner membrane (Alteri et al., 2011). Overall, more evidence is needed to confirm its impact on the cellular microenvironment and the potential involvement of other mechanisms.

**Table 1:** Effect of common efflux pump inhibitors on the colistin MICs.

Isolate	*mcr*	Efflux pump inhibitors				FC		References
		CCCP	NMP	PAβN	other	≥4	2	
*E. coli*	■	■	×	×	×	■	×	Liao et al. (2020)
*E. coli*	×	■	×	×	×	■	×	Osei Sekyere and Amoako (2017)
*K. pneumoniae*	■	■	×	×	×	■	×	Baron and Rolain (2018)
*K. pneumoniae*	×	■	×	×	×	■	×	Osei Sekyere and Amoako (2017)
*K. pneumoniae*	×	×	×	■	×	■	×	Sun L. et al. (2020)
*K. pneumoniae*	×	×	■	×	×	×	■	Naha et al. (2020)
*A. baumannii*	Unknown	■	×	×	×	■	×	Ni et al. (2016)
*A. baumannii*	×	■	×	■	TZ, CPZ	■	×	Machado et al. (2018)
*A. baumannii*	×	■	■	×	×	■	×	Yilmaz et al. (2020)
*A. baumannii*	×	■	×	×	DNP	■	×	Park and Ko (2015)
*P. aeruginosa*	×	■	×	×	×	■	×	Baron and Rolain (2018)
*P. aeruginosa*	Unknown	×	×	■	×	■	×	Ni et al. (2016)
*S. marcescens*	×	■	×	×	×	■	×	Baron and Rolain (2018)
*S. maltophilia*	Unknown	■	×	×	×	■	×	Ni et al. (2016)
*S. maltophilia*	Unknown	×	■	×	×	■	×	Ni et al. (2016)
*E. cloacae*	■	■	×	×	×	■	×	Baron and Rolain (2018)
*E. cloacae*	×	■	×	×	×	■	×	Osei Sekyere and Amoako (2017)
E. cloacae*	×	×	×	■	×	■	×	Telke et al. (2017)
*C. freundii*	×	■	×	×	×	■	×	Osei Sekyere and Amoako (2017)
*S. enterica*	×	■	×	×	×	■	×	Baron and Rolain (2018)
*M. morganii*	×	■	×	×	×	■	×	Baron and Rolain (2018)
*P. mirabilis*	×	■	×	×	×	■	×	Baron and Rolain (2018)
*A. hydrophila*	Unknown	×	×	■	×	■	×	Lo et al. (2022)
*B. intermedia*	×	■	×	×	×	■	×	Zoaiter et al. (2023)

TZ, thioridazine; CPZ, chlorpromazine; DNP, 2,4-dinitrophenol; FC, Fold change; *E. cloacae/E. asburiae.

**Figure 1:**
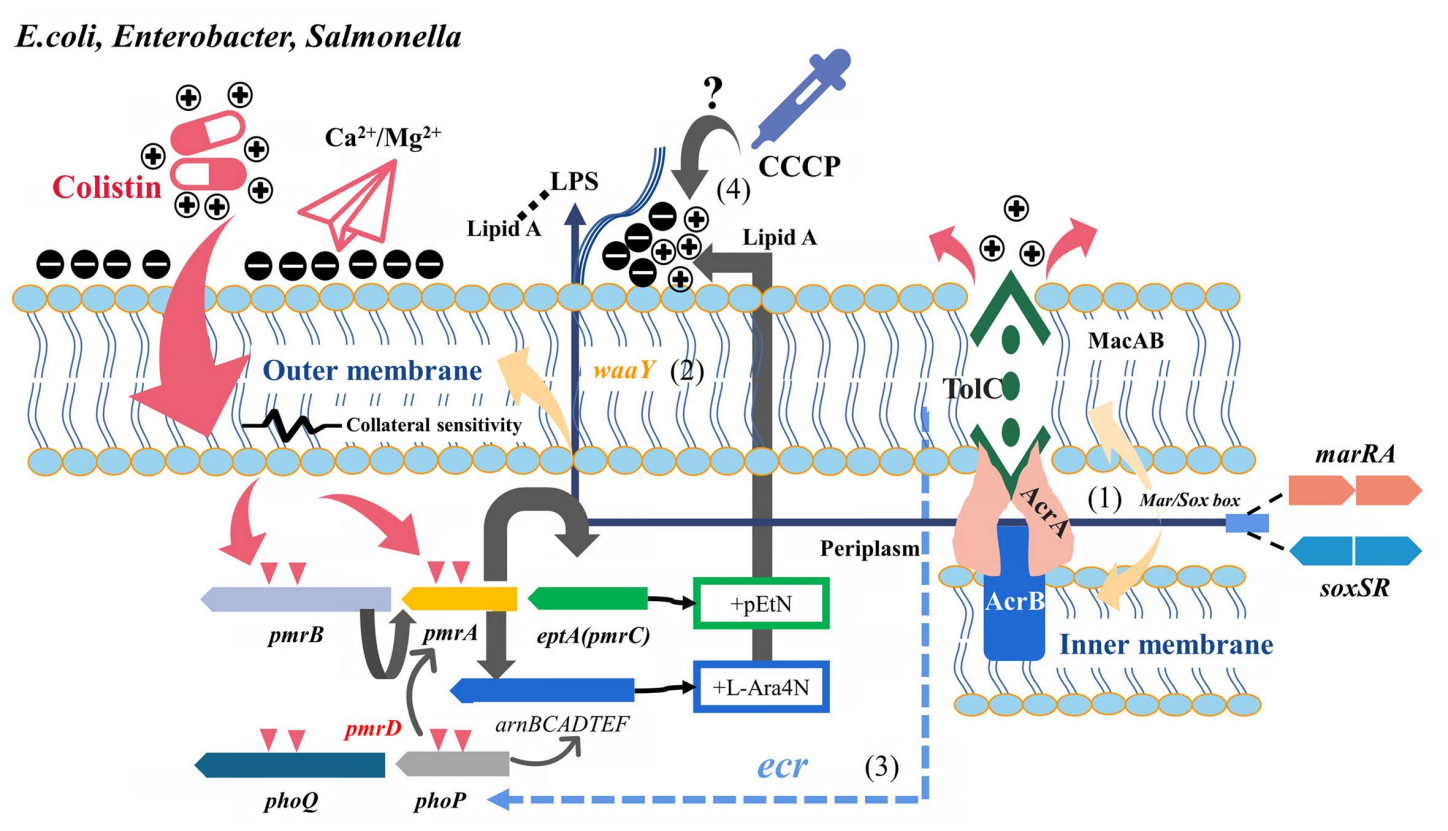
Potential relationship between multidrug resistance efflux pumps and their transcriptional regulatory factors and colistin resistance (*E.coli*, *Enterobacter*, and *Salmonella*). Two-component regulatory systems (PmrAB, PhoPQ, etc.) and related pathway variations are the classical pathways of *mcr*-negative colistin-resistant isolates. (1) Derepressed *marA/soxS* activated AcrAB-TolC or other RND efflux pumps involved in antibiotics efflux. (2) *marA* overexpression conferred collateral sensitivity to cationic antimicrobial peptides by up-regulating *waaY*. (3) Role of *tolC* in regulating PhoPQ via *ecr* in *Enterobacter* spp. remains to be confirmed. (4) Whether the effect of CCCP on the bacterial microenvironment mediates the change in colistin susceptibility is unclear.

Efflux pumps belonging to RND, MATE, SMR, and MFS families can use proton motive force to mediate efflux to various antibiotics (Lomovskaya and Watkins, 2001). Notably, CCCP reduces the activity of these multidrug-resistant efflux pumps by disrupting the proton motive force through interference with the transmembrane potential. A recent study found that CCCP, but not PAβN, reversed colistin resistance in a colistin-resistant *K. pneumoniae* strain that was *mcr*-negative and had no mutations in *mgrB*, *phoPQ*, *pmrABCDK* and reported efflux pump-related genes (*ramAR*, *acrAB*, *kpnEF*/GH, *soxS*, etc.) (Pu et al., 2023). It remains unclear whether other proton motive force-dependent and energy-driven efflux pumps contribute to colistin resistance (Figure 1). Additionally, the *crrB* mutant *K. pneumoniae* had a 4-fold decreased for colistin MIC in the presence of PAβN, suggesting a potential role of the efflux pump in colistin resistance (Sun L. et al., 2020). In the presence of NMP, the colistin MICs in colistin-resistant with *mcr*-negative *A. baumannii*/K have been observed*. Pneumoniae* lacking lipid A-related modification gene variation decreased significantly (Naha et al., 2020; Yilmaz et al., 2020). Li et al. also found that the colistin MICs in ST11-*bla*_KPC-2_ resistant lineage significantly reduced in the presence of NMP (Yang et al., 2021). Although these results suggest a potential pathway for the involvement of efflux pumps in colistin resistance, more substantial evidence is still needed. Considering the differences among species, we then summarized in detail the roles of the efflux pumps and their related genes in colistin resistance in different species.

## Enterobacteriaceae

### *Escherichia coli*, *Enterobacter*, and *Salmonella*

Colistin-resistant Enterobacteriaceae are widely identified in clinical infection settings and animal husbandry, and the status of antimicrobial resistance is a concern (Shen et al., 2019). As a transcription regulator commonly found in *E. coli*, *K. pneumoniae*, and other members of Enterobacteriaceae, *marR* played an essential role in regulating efflux pumps. Furthermore, *marR* mediated the inhibition of *marA*, while salicylic acid, antibiotic pressure, and endogenous amino acid replacement could release the disinhibition and activate *marA* expression under physiological conditions, thus upregulating the MDR efflux pump AcrAB-TolC (Grkovic et al., 2002). Activated *marA* could also upregulate the expression of gene encoding lipopolysaccharide core heptose (II) kinase, known as *waay*. This upregulation increases the negative charge of the bacterial outer membrane, subsequently enhancing its ability to bind cationic antimicrobial peptides (collateral sensitivity) (Lázár et al., 2018). Sundaramoorthy et al. (2019b) found that the negative charge of the outer membrane of colistin-resistant *E. coli* increased in the presence of the *marR* inhibitor salicylic acid, thereby restoring susceptibility to colistin. Another study found that the decreased polymyxin B susceptibility due to the *marAB* upregulation and *marR* mutant was observed, mainly attributed to the action of AcrAB and other TolC-dependent efflux pumps (Warner and Levy, 2010). These findings demonstrated the different pathways through which *marRA* is involved in colistin resistance. Another *soxRS* system, common in *E. coli* and *Salmonella*, plays an essential role in coping with superoxide, nitric oxide, and antibiotic stress. Increased *soxS* expression induced multidrug-resistant efflux pump AcrAB expression, decreased membrane permeability, and promoted the formation of multidrug-resistant phenotypes (Koutsolioutsou et al., 2005). The same binding sites exist in the promoters regulating Sox, Rob, and Mar systems (*mar*/*sox* box), resulting in a high degree of overlap between the genes (*micF*, *acrAB*, *ompF*, *fumC*, etc.) of *soxRS* and *mar* regulons (Figure 1) (Martin et al., 1999). Both *soxRS* and *waaY* could be activated by superoxide induction, but *waaY* transcription was *SoxRS* dependent (Figure 1) (Lee et al., 2009). Although the absence or overexpression of *soxS* in *E. coli* harboring the *soxR* mutation did not significantly affect CAMP susceptibility, the overexpression of *SoxS* in the *tolC* mutant contributed to increased susceptibility to polymyxin B (Warner and Levy, 2010). A recent study showed that not only the collateral susceptibility of antimicrobial resistance development to cationic polypeptides was associated with the changes in the regulation of LPS-related genes, but also a variety of physiological metabolism-related genes such as iron ion-binding proteins and transmembrane transport proteins played an important role (Grézal et al., 2023). This also reflected the pleiotropy of these global regulators involved in regulating multiple links of physiological metabolism. The disruption of *tolC* directly affected outer membrane integrity, and the intracellular accumulation of toxic substances activated *marA*, *soxS*, and *rob* to upregulate the efflux pumps (Rosner and Martin, 2009; Zgurskaya et al., 2011). Additionally, Rob might directly or indirectly increase *marRAB* expression by upregulating *micF* in response to colistin pressure, although the *rob* deficiency had little effect on CAMP susceptibility (Oh et al., 2000; Warner and Levy, 2010).

Heteroresistance may be an intermediate stage in the transition of susceptible bacteria to antimicrobial resistance, which is an essential reason for the failure of clinical anti-infective therapy (Falagas et al., 2008). Telke et al. found that *soxRS*-induced AcrAB-TolC efflux pump mediated heterogeneous resistance to colistin and could restore colistin susceptibility through PAβN in *Enterobacter* spp. (Telke et al., 2017). Unlike CCCP and DNP, PAβN could act directly as an inhibitor of AcrAB and AcrEF efflux pumps. Therefore, these results also suggested that AcrAB efflux pump was involved in colistin resistance (Misra et al., 2015). Colistin could mediate bactericidal effects by producing hydroxyl radicals or causing oxidative stress reactions as a class of bactericidal antibiotics (Bialvaei and Samadi Kafil, 2015; Yu et al., 2019). However, this bactericidal effect did not appear to influence the *soxRS* transcriptional levels in heterogeneous colistin-resistant *Enterobacter* isolates (Telke et al., 2017). The necessity of colistin heteroresistance by *tolC* (but not *acrB*) was further confirmed in *E. cloacae*, and *tolC* could activate the PhoQ-PhoP system by affecting the *ecr* (Figure 1) (Huang et al., 2019). The deletion of *acrB* is not sufficient to completely reverse colistin heterologous resistance in *E. cloacae* (Huang et al., 2019). Previous findings suggested efflux pumps are the primary mechanism for generating stable heterogeneous subpopulations (Manjunath et al., 2021). The fitness costs of these resistance mechanisms remain to be evaluated. Besides the co-regulation of *tolC* with Mar, Rob, and Sox, the expression of *tolC*, which carried multiple promoters, was also affected by EvgAS and PhoPQ, indicating that AcrAB-TolC had the potential to cope with different environments and responded quickly (Zhang et al., 2008; Pérez et al., 2012).

Transcriptome and proteomics analyses revealed that the expression of efflux pump AcrAB in *E. coli* and *K. pneumoniae* significantly increased after exposure to polymyxin B under experimental conditions (Ramos et al., 2016; Liu et al., 2020). The overexpression of *cpxR* in the absence of *acrB* affected the expression of TCS genes (*phoP*, *phoQ*, *pmrB*, *pmrC*, *pmrH*, and *pmrD*) and restored colistin susceptibility in *Salmonella* (Zhai et al., 2018). The variation in the *acrB* 620 site in *Salmonella* contributed to the restoration of colistin susceptibility, which could be explained by the defective macromolecular transport due to the altered conformation of the switch loop in this site (Cha et al., 2014; Kapach et al., 2020). Either *tolC* deletion alone or dual deletion of *cpxR* and *tolC* increased the susceptibility of *S. enterica* to colistin to varying degrees. The inactivation of *tolC* impaired the function of the entire RND efflux family and other *tolC*-dependent efflux pumps, not only the AcrAB pump (Zhang M. K. et al., 2021). These results highlighted the different contributions of *acrB*, *soxRS*, and *marRA* to colistin resistance in different backgrounds, the role of *tolC* mutations in regulating colistin resistance, and the complex regulatory network among the efflux pump, two-component system, and LPS modification.

### Klebsiella pneumoniae

**Figure 2:**
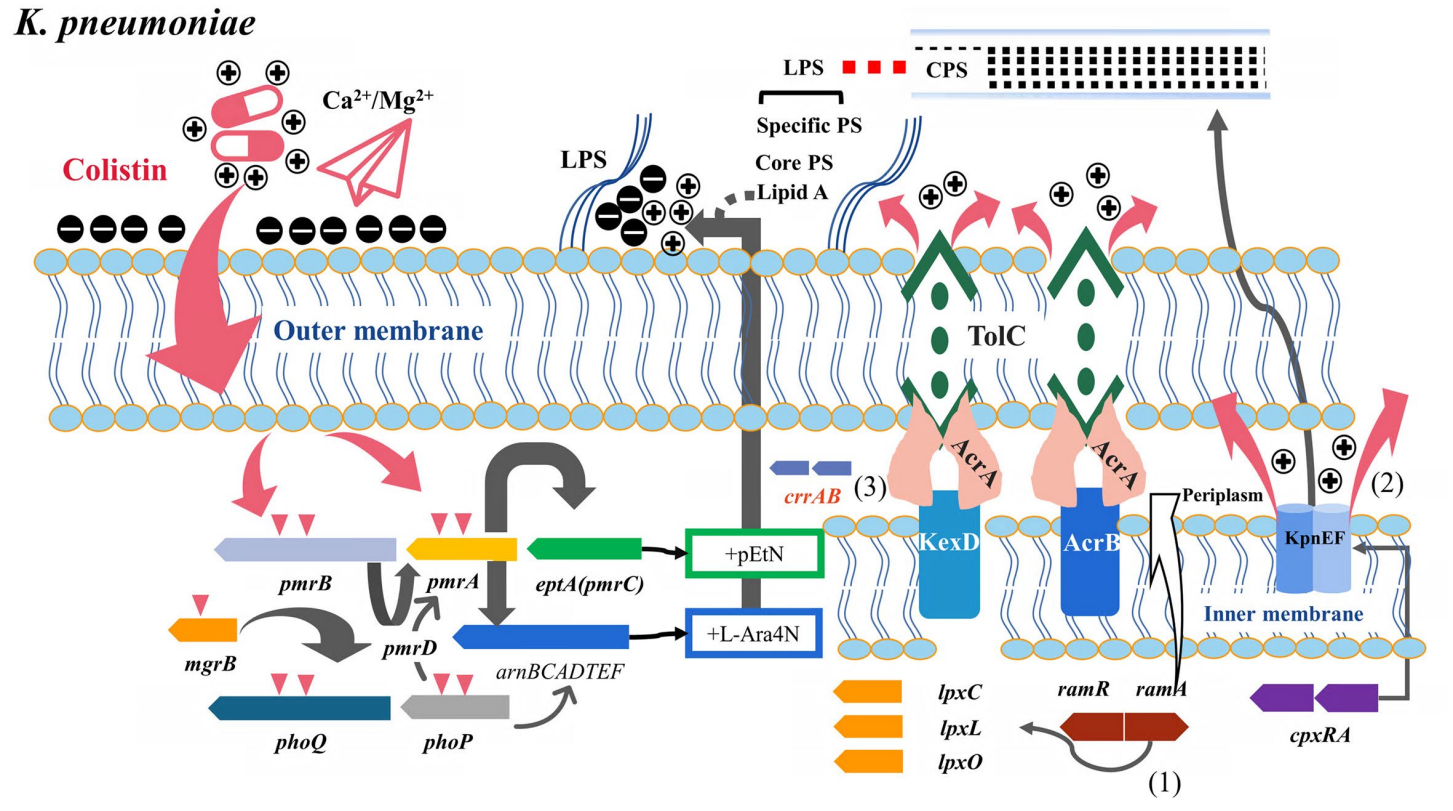
Potential relationship between multidrug resistance efflux pumps and their transcriptional regulatory factors and colistin resistance (*K. pneumoniae*). (1) *ramAR* was the dominant efflux pump transcriptional regulator in *K. pneumoniae*, binding to *lpxC/X/O* and participating in lipopolysaccharide synthesis. (2) KpnEF efflux pump was regulated by *cpxRA* and involved in colistin efflux and capsule synthesis. (3) Co-expression of CrrAB with the adjacent KexD efflux pump promoted the development of high levels of colistin resistance.

Multicenter studies from China have shown that the insertion inactivation of *mgrB* is the primary mechanism of colistin resistance in *K. pneumoniae* (Li et al., 2023). However, *acrR* insertion by IS*26* is widespread in ST11-*bla*_KPC-2_-producing PR-CRKP (Polymyxin-resistant carbapenem-resistant *K. pneumonia*), which may be related to the low level of colistin resistance in these high-risk clones (Yang et al., 2021; Li et al., 2023). *ramRA* is a widely described global regulator in *K. pneumoniae* compared with *E. coli* and *Salmonella*, which mediates resistance to antibiotics such as tigecycline, nitrofurantoin, and beta-lactams by regulating AcrAB and OqxAB (Hao et al., 2022). The expression of *soxS*, *ramA*, and *acrAB-tolC* significantly upregulated in *mcr*-negative colistin-resistant *K. pneumoniae* lacking specific TCS-related gene variants, suggesting the role of *soxS*, *ramA*, and efflux pumps in colistin resistance (Naha et al., 2020). Similar to *marA* and *soxS*, *ramA* can also affect LPS modification. *ramA* directly binds to and activates the genes involved in lipid A biosynthesis: *lpxC*, *lpxL*-2, and *lpxO*, thereby modifying lipid A and resulting in decreased colistin susceptibility and increased anti-serum phagocytosis under the condition of increased *ramA* expression (Figure 2) (De Majumdar et al., 2015). Li et al. demonstrated *in vitro* that *ramR* variants can mediate polymyxin resistance, possibly related to derepressed *ramA*-mediated LPS alterations and multidrug resistance efflux pump overexpression (AcrAB) (Li et al., 2023). Another survey in China showed that the transcription level of *ramA* in colistin-resistant *K. pneumoniae* was not statistically significantly different from that in non-colistin-resistant strains, but the study lacked the main antimicrobial resistant and hypervirulent lineages ST11 and ST23 prevalent in China (Wang et al., 2017). However, *ramA* overexpression and variation were common in some colistin-resistant *K. pneumoniae* ST11 and ST147 isolates (Naha et al., 2020; Lv et al., 2021; Bolourchi et al., 2021a,b). Cationic antimicrobial peptides are also common in the human environment. Thus, whether these high-risk clones contribute to the co-evolution of hypervirulence and antimicrobial resistance by activating the efflux pump is not clear. A previous study showed that Δ*acrB K. pneumoniae* mutant had increased susceptibility to colistin and that LPS and CPS production were not significantly affected, revealing a role for AcrAB in the antimicrobial peptides resistance (Padilla et al., 2010). A less-described class of multidrug efflux RND transporter, KexD, is also present in *K. pneumoniae* and can be expressed constitutively with *E. coli* TolC or *K. pneumoniae* KocC (Ogawa et al., 2012). *KexD* has been proven to contribute to colistin resistance, and both of them are more conducive to high-level colistin resistance in *K. pneumoniae* under the induction of its neighbor gene *crrB* (Figure 2) (Cheng et al., 2018; Pantel et al., 2023). The specific diversity of the *crrBAC*-*kexD* cluster in the *K. pneumoniae* ST11 group suggests that ST11, a closely related lineage with carbapenem resistance and hypervirulence, is at risk of further developing colistin resistance (Kim et al., 2022). Overall, the colistin resistance mechanisms in *mcr-*negative colistin-resistant pathogens may be accumulated, highlighting the synergistic involvement of multiple mechanisms. Notably, the homolog of *mexCD*-*oprJ* efflux, *tmexCD*-*toprJ*, can be plasmid-mediated to acquire resistance to tigecycline in *Enterobacteriaceae* rapidly (Lv et al., 2020). In recent years, it has been identified that *tmexCD-toprJ* and *mcr* coexist in the same host of mobile elements, declaring the failure of last-line antibiotics (tigecycline and colistin) treatment and suggesting the threat of the rapid emergence of superbugs (Sun S. et al., 2020; Dong et al., 2022).

A study showed that several *mcr*-negative colistin-resistant *K. pneumoniae* strains isolated from CC15 and CC101 had different degrees of overexpression of *acrAB*, *ramA*, *kpnEF* (SMR pump), and *kpnGH* (MFS pump) (Naha et al., 2022). *KpnEF* efflux pumps belong to the small MDR family. The *Δ*KpnEF mutants showed increased susceptibility to various cationic antimicrobial peptides such as colistin, whereas *KpnEF* expression differs in the *cpxAR* mutant background (Figure 2) (Srinivasan and Rajamohan, 2013). The *kpnGH* is homologous to *emrAB* and belongs to the MFS efflux pump. *In vitro*, *Δ*kpnGH mutant susceptibility to cephalosporins, imipenem, polymyxin B, chlorhexidine, and other antimicrobial agents increased (Srinivasan et al., 2014).

### Acinetobacter baumannii

The antimicrobial resistance surveillance from China showed that the colistin resistance rate of *A. baumannii* was still relatively low, but more than half of the isolates showed carbapenem-resistant *A. baumannii* (CRAB) (Gao et al., 2017; Zhang et al., 2020). *A. baumannii* had intrinsic resistance to multiple antibiotics. Hence, colistin has become one of the few options for treating CRAB. In the clinical application of colistin in anti-infection therapy, it is easy to induce *A. baumannii* to lead to colistin resistance by modifying phosphoethanolamine mediated by the two-component regulatory system PmrAB (Qureshi et al., 2015). In *A. baumannii*, three significant efflux pumps, AdeAB, AdeIJK, and AdeFGH, are involved in expelling antibiotics such as tetracycline, beta-lactam, and quinolones (Ayoub Moubareck and Hammoudi Halat, 2020). After *A. baumannii* was exposed to colistin, the upregulation of Ade cluster encoding genes was common; *emrB* and *macAB* overexpression were also observed (Cheah et al., 2016; Hua et al., 2017; Boinett et al., 2019). In *E. coli*, MacA demonstrated high affinity and specificity for the core LPS, suggesting that MacAB-TolC could be involved in LPS transport (Lu and Zgurskaya, 2013). *phoP* inhibited *macAB* transcription, and *macAB* deletion attenuated *Salmonella*’s virulence more than the *tolC* mutant (Nishino et al., 2006). Despite the upregulation of MDR efflux pumps, the laboratory-induced colistin-resistant isolates showed restoration of susceptibility to cefepime, azithromycin, and teicoplanin compared with the parental isolates (Moffatt et al., 2010; Li et al., 2015). Hua et al. also found that colistin induced partial restoration of antibiotic susceptibility in resistant strains but they mainly focused on β-lactams (Hua et al., 2017). Increased susceptibility to bacitracin, vancomycin, and beta-lactams was also observed in *A. baumannii* with high permeability of the outer membrane (Leus et al., 2018). These phenomena suggested that efflux pumps might be more involved in the transport of toxic compounds rather than in the efflux of the dominant antibiotics in colistin-resistant strains with increased outer membrane permeability due to LPS loss (Henry et al., 2012; Henry et al., 2015). Such collateral sensitivity could be masked by multiple β-lactamases and other plasmid-mediated resistance determinants in some clinical isolates of colistin-resistant *A. baumannii*.

Machado et al. (2018) identified several heterogeneous colistin-resistant strains that lacked TCS gene variants (*lpxACD* and *pmrCAB*). The EPIs CCCP, NMP, and PAβB were found to reverse polymyxin resistance to varying degrees in these strains. Furthermore, these strains’ efflux pump genes *adeB*, *adeJ*, *adeG*, *craA*, *amvA*, *abeS*, and *abeM* were overexpressed after colistin exposure (Machado et al., 2018). Colistin resistance mediated by the mutations in the *pmr* operon is generally expensive for *A. baumannii* (Geisinger and Isberg, 2017). Colistin heteroresistant *A. baumannii* isolates with only *lpxACD* mutations and overexpression of *adeAB*, *adeG*, and *adeIJK* were also observed in other studies, highlighting the role of efflux pumps in colistin heteroresistance (Chen et al., 2020). Besides participating in antibiotics efflux, the overexpression of these efflux pumps may be associated with fitness advantages mediated by these clinical isolates at specific sites of infection (Yoon et al., 2016). Another study also demonstrated that the resistance of *A. baumannii* to colistin could be reversed by NMP. AdeRS mutations mediating AdeAB or other RND-type efflux system overexpression were suggested to be the possible cause of colistin resistance in these isolates (Yilmaz et al., 2020). Additionally, increased expression of efflux transporter proteins (AdeABC and HlyD family) after colistin exposure was also observed in another study (Cheah et al., 2016). However, neither AdeRS nor AdeAB was shown to affect the colistin MIC *in vitro* (Richmond et al., 2016). These findings also suggested that efflux pump overexpression played a role in the excretion of toxic compounds and maintaining outer membrane integrity besides antibiotic efflux. The role of efflux pumps in antimicrobial resistance is also influenced by specific physiological settings (Leus et al., 2018). Additionally, different biological effects were found in AdeB deletion strains with different genetic backgrounds, suggesting that the function of the AdeAB efflux pump might be heterogeneous in other individuals (Richmond et al., 2016). These results indicated that the AdeAB pump could target different substrates under different living environments and selective pressures.

**Figure 3:**
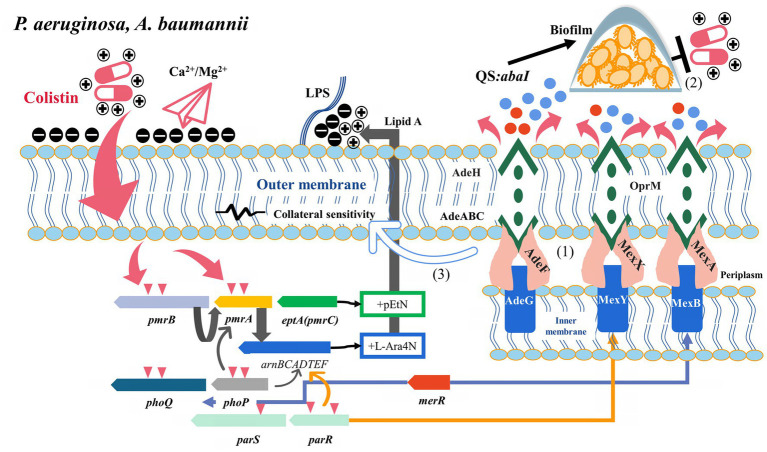
Potential relationship between multidrug resistance efflux pumps and their transcriptional regulatory factors and colistin resistance (*P. aeruginosa* and *A. baumannii*). (1) AdeRS, *merR*, and *parRS* activated AdeAB, MexXY-OprM, and MexAB-OprM to efflux antibiotics, respectively. (2) Efflux pumps were involved in colistin tolerance by transporting signaling molecules to activate the quorum sensing system. (3) Some colistin-resistant *A. baumannii* strains with efflux pump overexpression exhibited increased susceptibility to multiple antibiotics, which is associated with the disruption of LPS.

The two-component system and efflux pumps can be activated in bacteria by pH, nutrients, redox state, osmotic pressure, quorum signaling, and antibiotics (Worthington et al., 2013; Bazyleu and Kumar, 2014). One reported that the expression of AdeRS, AdeABC, and AdeFGH promoters did not show significant differences under sub-MIC colistin concentration and different growth conditions (Gil et al., 2021). However, the activation of the efflux pumps *adeB*, *adeG*, *adeJ*, *adeH*, and autoinducer synthase (*abaI*) by subinhibitory colistin has been observed in some clinical isolates (Sato et al., 2018; Shenkutie et al., 2022). Multiple biological effects on bacteria are observed at sublethal antibiotic concentrations, one of which can directly engage or interfere with quorum sensing systems (QS) (Figure 3) (Andersson and Hughes, 2014). As a signal molecule in the QS system, *abaI* is essential in regulating biofilms and can be used as a substrate for the AdeFGH efflux pump (He et al., 2015). *A. baumannii* isolates in a biofilm state has higher minimal biofilm inhibition concentrations (MBICs), which helps reduce the therapeutic effect of colistin (Kim et al., 2015). However, AdeRS did not always exist in *A. baumannii* isolates (Montaña et al., 2015). The restoration of colistin susceptibility by EPIs was observed in some colistin-resistant isolates without *adeRS*, suggesting the existence of other regulatory pathways and efflux pumps involved in colistin resistance (Yilmaz et al., 2020). Lin et al. observed that the transcription of *emrB* and several *emrB*- like genes were upregulated in colistin resistance-induced *A. baumannii*. Furthermore, Δ*emrB* mutants had increased susceptibility to colistin, demonstrating the contribution of the EmrAB pump to colistin resistance in *A. baumannii* (Lin et al., 2017). EmrAB was previously described mainly in *E. coli* and could mediate increased resistance to nalidixic acid, thiolactamycin, nitroquinoline, and hydrophobic proton uncouplers, with relatively little information available in *A. baumannii* (Yousefian et al., 2021). In another study, significant transcriptional changes were observed in the MATE (*ydhE*), MFS (*mdfA*), and SMR (*ynfA* and *sugE*) efflux pumps of colistin-resistant *A. baumannii* upon exposure to subinhibitory colistin concentrations (Paul et al., 2020). The overexpression of the MATE family efflux pump and *mdfA* contributes to the efflux of cationic compounds, but their contribution to colistin resistance remains confirmed (He et al., 2011; Li et al., 2015). A putative solvent/toluene-tolerant efflux ABC transporter protein, Ttg2C, may be essential in high-level colistin resistance in *A. baumannii* (Thi Khanh Nhu et al., 2016).

### Pseudomonas aeruginosa

*Pseudomonas aeruginosa* is a common pathogen causing burn infection and cystic fibrosis, exhibiting intrinsic resistance to different antibiotics. MexAB-OprM, MexXY-OprM, and MexCD-OprJ are widely described RND family efflux pumps in *P. aeruginosa* with various efflux substrates: β-lactams, aminoglycosides, quinolones, tetracyclines, tigecycline, macrolides, amphenicols, novobiocin, sulfonamides, and trimethoprim (Li et al., 2015). MexXY is an induced efflux system, often characterized by concentration-dependent induction by ribosomal inhibitors (such as chloramphenicol, tetracyclines, macrolides, and aminoglycosides) (Jeannot et al., 2005). However, the overexpression of MexXY under exposure to ribosome-targeting antimicrobial agents was inversely correlated with colistin susceptibility and accompanied by the downregulation of the *arn* operon (Poole et al., 2015). Meanwhile, the heterogeneity of MexXY expression was observed in clinically isolated colistin-resistant *P. aeruginosa* isolates with different resistance levels, suggesting the possibility of intervention by other mechanisms (Goli et al., 2016). In an experiment on chlorhexidine (cationic polypeptide compounds) induced resistance, several mutants with decreased colistin susceptibility were observed, and these mutants exhibited MexY overexpression. While adding chlorpromazine significantly reduced the chlorhexidine MICs in the resistant mutants (Tag ElDein et al., 2021). Under colistin selective pressure, the two-component regulator ParR-ParS activated the *arnBCADTEF* operon, promoted *mexY* overexpression and inhibited *oprD* to reduce susceptibility to colistin (Fernández et al., 2010; Muller et al., 2011). *mexXY* deletion in ParRS-dependent pathways increased colistin tolerance by upregulating *arnA* and *pmrA* expression. The simultaneous overexpression of the *arn* opern and *mexXY* induced by the dual activation of PmrAB and ParRS contributed to the high-level resistance of the *pmrB* mutants to colistin, suggesting that the synergistic effect of efflux pumps and LPS modification promoted the development of colistin resistance (Puja et al., 2020) (Figure 3). In another study on the development of cross-resistance to colistin by exposing *P. aeruginosa* to chlorhexidine, proteomics revealed that the upregulation of MexA expression might be related to colistin resistance (Hashemi et al., 2019). Zhang W. et al. (2021) also observed significant upregulation of *mexAB*-*oprM* in laboratory-induced colistin-resistant *P. aeruginosa*. These results were similar to the previous findings of Pamp et al., who found that MexAB was involved in colistin tolerance, especially in adapting different subpopulations of bacteria to colistin in *P. aeruginosa* biofilms (Pamp et al., 2008). The efflux pump activator MerR induced mexAB-oprM and mexEF-oprN to participate in biofilm tolerance and acted as a repressor of *phoPQ* to participate in colistin resistance (Chambers and Sauer, 2013). In *P. aeruginosa*, the efflux pump is involved in the transport of signal molecules, and its expression can also be affected by the QS system. The genes related to QS were upregulated when *P. aeruginosa* was exposed to subinhibitory colistin concentration (Figure 3) (Cummins et al., 2009). However, whether this pathway can promote colistin resistance by activating efflux pumps or biofilms remains to be confirmed.

*rsmA* is a post-transcriptional regulatory protein involved in regulating various virulence-related genes, and its deletion causes overexpression of the MexEF-OprN pump and downregulation of type III secretion (Burrowes et al., 2006). However, the disruption of type III via secretion *rsmA* is associated with the overexpression of MexCD-OprJ or MexEF-OprN (Linares et al., 2005; Mlynarcik and Kolar, 2019). On exposure to different membrane-targeted drugs, these strains can mobilize various genetic determinants, such as *pmr* operons and efflux pumps, in response to environmental stresses (Chiang et al., 2012). The MexAB-oprM, MexCD-oprJ, and MuxABC-opmB efflux pumps all contribute to colistin-tolerant subpopulations (Chiang et al., 2012).

### Stenotrophomonas maltophilia

*Stenotrophomonas maltophilia* is widespread in the natural environment and exhibits natural resistance to numerous antibiotics, significantly limiting clinical use options. Despite colistin’s *in vitro* antimicrobial activity, the assessment of colistin susceptibility in *S. maltophilia* is influenced by various factors *in vitro*. (Martínez-Servat et al., 2018). Lin et al. found that MacABCsm had a broader substrate spectrum on macrolides, aminoglycosides, and polymyxin than its counterpart in *E. coli*, and the deletion of MacAB resulted in a significant decrease in the colistin MIC of *S. maltophilia* isolates (Lin et al., 2014). Meanwhile, MacABCsm was stably expressed in *S. maltophilia*, associated with intrinsic resistance (Lin et al., 2014). Although multiple genes encoding efflux pumps have been identified in colistin-resistant *S. maltophilia*, their specific contribution to colistin resistance remains elucidated (Li et al., 2019).

### Aeromonas hydrophila

*Aeromonas hydrophila* is a common group of opportunistic pathogens in the *Aeromonas* genus associated with aquatic environments. The AheABC efflux pump regulates the efflux of cefoperazone, cefuroxime, erythromycin, pristinamycin, and trityltin in *A. hydrophila* and participates in its MDR phenotype (Hernould et al., 2008). A recent study showed that the expression levels of three putative RND efflux pump genes, AHA0021, AHA1320, and AheB, significantly increased in MDR *A. hydrophila*. Also, PAβN significantly reduced the MIC of piperacillin/tazobactam, imipenem, erythromycin, and polymyxin B, suggesting the contribution of the RND efflux pump to colistin resistance (Lo et al., 2022).

## Efflux pumps are associated with intrinsic colistin resistance in other gram-negative bacteria

### *Serratia marcescens* and *Proteus mirabilis*

*Proteus* and *S. marcescens* are also common but easily overlooked opportunistic pathogens in clinical infections, often showing inherent resistance to colistin. LPS modification mediated by L-Ara4N in lipid A, Kdo residues, and *arnBCADTEF* operon contributes to the intrinsic resistance of *P. mirabilis* and *S. marcescens* to colistin (Olaitan et al., 2014). A recent study showed that the ABC transporter MacAB contributed to the intrinsic colistin resistance of *S. marcescens*, which was previously thought to be associated with the efflux of macrolide antibiotics and could be constitutively expressed with *tolC* (Shirshikova et al., 2021). In *Salmonella*, *phoP* inhibited *macAB* transcription, and *macAB* deletion attenuated the virulence of *Salmonella* more than the *tolC* mutant (Nishino et al., 2006). LPS played a role in colistin resistance and was a critical virulence factor in pathogenesis. However, whether *macAB* in *S. marcescens* is associated with PhoPQ or other TCSs remains to be investigated. The MFS efflux pump family SmvA was overexpressed in *K. pneumoniae* with increased resistance to multiple cationic biocides (chlorhexidine and octenidine) (Wand et al., 2019). Another study on *P. mirabilis* demonstrated that SmvA expression was insufficient to explain the differences in polymyxin B MIC of these intrinsically resistant isolates (Pelling et al., 2019).

### *Neisseria* spp.

Among *Neisseria* spp., *N. meningitidis* and *N. gonorrhoeae* are closely associated with clinical infections and causative agents of bacterial meningitis and gonorrhea, respectively. The MtrCDE efflux system in *Neisseria* is thought to be the leading cause of the low-level intrinsic resistance to colistin (Moffatt et al., 2019). MtrCDE is Neisseria’s most widely explored RND pump, contributing to resistance to β-lactams, macrolides, rifampicin, detergents, bile salts, and cationic polypeptides. The contribution of MtrCDE to colistin resistance has been demonstrated *in vitro* (Tzeng et al., 2005). The epistatic effects between *mtrD* and *mtr* promoter region promote the formation of the multidrug resistance phenotype (Wadsworth et al., 2018).

### *Yersinia* spp.

*Yersinia* comprises dozens of species, and only *Y. pestis*, *Y. pseudotuberculosis*, and *Y*. *enterocolitica* are closely related to clinical infection. Significantly different from other bacteria, the resistance of *Yersinia* to cationic peptides (including colistin) is affected by temperature as well as individual differences, which may be related to the successful adaptation of these pathogens at different sites (Bengoechea et al., 1996, 1998). Bengoechea et al. found that the *rosAB* locus encoded a temperature-regulated efflux pump and could participate in colistin efflux in response to antibiotic pressure (Bengoechea and Skurnik, 2000).

### Burkholderia

Like *S. maltophilia*, *Burkholderia* is widespread in the environment and exhibits inherent resistance to various antibiotics, including colistin. The primary mechanism of intrinsic resistance of *Burkholderia* to colistin is still Ara4N synthesis and Ara4N transfer to lipid A (Loutet and Valvano, 2011). A previously suggested *norM* belonging to MATE transporters contributes to colistin resistance, but it is mainly associated with the presence of tetracycline (Fehlner-Gardiner and Valvano, 2002). Another class of *yej* operons (*yejA1*, *yejA2*, *yejB*, *yejE*, and *yejF*) belonging to ABC transporters was also found to be directly activated by colistin and conferred colistin resistance. To further clarify the contribution of the efflux pump to colistin resistance, Zoaiter et al. (2023) found that CCCP restored susceptibility in *Burkholderia* isolates, while VRP, PAβN, and RSP did not. The genomic analysis showed that the efflux pump genes YejABEF, LolCDE, and NorM were widely present in these isolates (Zoaiter et al., 2023). This result suggested that multiple efflux pumps might be involved in the intrinsic colistin resistance of *Burkholderia*. Another study explored the interaction of the Amrab-OprA, BpeEF-OprC, and BpeAB-OprB efflux pumps with the outer membrane permeability and showed that only the simultaneous presence of Amrab-OprA inactivation and hyperporination contributed to the increased susceptibility to colistin (Krishnamoorthy et al., 2019).

## Future perspectives

Compared with the two-component regulatory system PmrAB/PhoPQ and *mcr*-mediated colistin resistance, relatively little information is available on the role played by efflux pumps in this regard. Although these findings suggest the possibility of EPIs reversing polymyxin resistance, EPI application to reverse colistin resistance still needs to be confirmed. CCCP has advantages over other EPIs in reversing colistin resistance, and its cytotoxicity limits its clinical application. Therefore, identifying these atypical resistant strains and developing suitable EPIs are crucial. Whether EPIs can reverse polymyxin resistance mediated by polymyxin-degrading protease and RpoE stress response pathways not involving structural changes in lipid A is uncertain. On the contrary, various reports showed that the bactericidal effect of CCCP on colistin was different. Therefore, the impact of CCCP on the bacterial intracellular microenvironment remains to be clarified. Other efflux pumps that rely on proton power to mediate colistin resistance remain to be identified. Since efflux pumps and associated transcription factors play a role in response to environmental stress, their activation can provide an adaptive advantage for these antibiotics-resistant strains in specific environments (Holden and Webber, 2020). However, the fitness effects of efflux pumps and related transcription factors mediating colistin resistance are still less explored compared with those of colistin resistance caused by LPS-related locus variants. The role of efflux pumps and their regulators in LPS synthesis, transport, and outer membrane integrity in colistin-resistant strains with different genetic and living backgrounds remains to be determined.

## Conclusion

The efflux pumps’ structure, function, and regulation have been previously summarized in detail in many comprehensive reviews (Piddock, 2006; Puzari and Chetia, 2017; Nishino et al., 2021). We specifically examined the effects of efflux pumps, their regulators, and EPIs on colistin susceptibility. Efflux pumps play a role in the classical pathway of antibiotic resistance and participate in the efflux of various metabolites and signaling molecules. The correlation between efflux pumps and their transcriptional regulators and LPS modification/transport indicates colistin resistance mechanisms’ complexity. The observation that efflux pumps and their regulator (*ramR*, KpnEF, KexD, etc.) independently mediate colistin susceptibility in some isolates indicates colistin resistance mechanisms’ diversity. The effect of efflux pumps on colistin susceptibility is also mediated through multiple pathways, such as heteroresistance and tolerance. These findings indicate that the multidrug resistance efflux pumps may participate in different stages of occurrence and development of colistin resistance. Overall, the contribution of efflux pumps and their regulators to colistin resistance is multi-pathway, including outer membrane permeability, LPS modification, and environmental adaptation, besides the direct involvement in efflux. For developing adjuvants acting as EPIs, it is critical to identify colistin-resistant strains that are *mcr*-negative and lack specific mutations related to LPS modification.

## Author contributions

YD, JH, WX, and ZZ contributed to the conception and design of the study. WX, XX, and CJ collected and explored literature. JH, MT, and GL performed the diagrams visualization. YD, ZZ, CY, JH, and JL prepared and revised the manuscript. All authors contributed to the article and approved the submitted version.

## Funding

This work was supported by the Sichuan Science and Technology Program (2020YFQ0045 and 2021YFS0329) and School-level scientific research project of Southwest Medical University (2019ZQN017).

## Conflict of interest

The authors declare that the research was conducted in the absence of any commercial or financial relationships that could be construed as a potential conflict of interest.

## Publisher’s note

All claims expressed in this article are solely those of the authors and do not necessarily represent those of their affiliated organizations, or those of the publisher, the editors and the reviewers. Any product that may be evaluated in this article, or claim that may be made by its manufacturer, is not guaranteed or endorsed by the publisher.

## Footnotes

^1^https://www.ncbi.nlm.nih.gov/pathogens/refgene/#mcr
