# Supplementary figures and images for: Role of efflux pumps, their inhibitors, and regulators in colistin resistance
Source: Front Microbiol. 2023 Aug 4;14:1207441. doi: 10.3389/fmicb.2023.1207441 (PMC10436536; doi:10.3389/fmicb.2023.1207441)

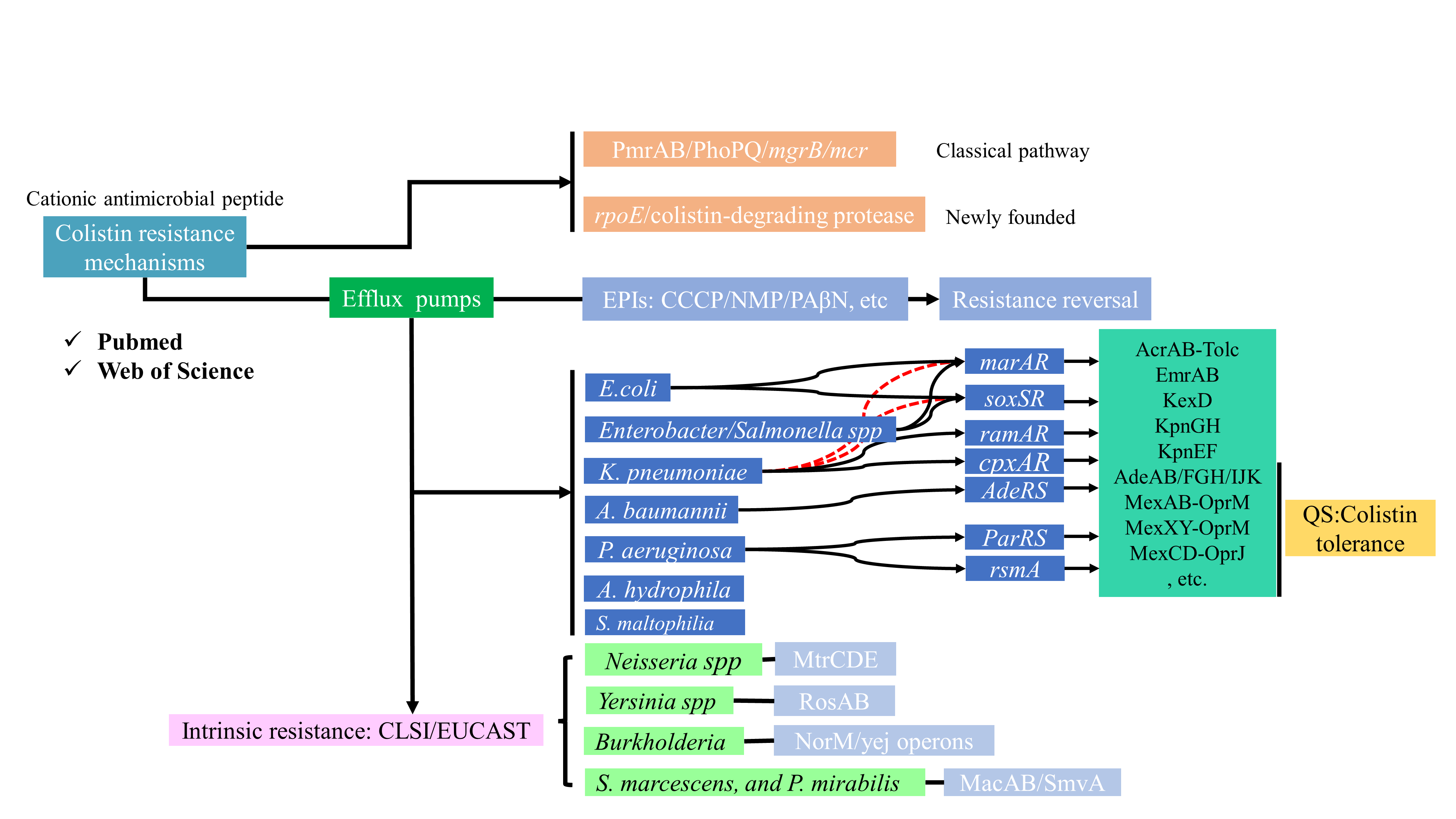

Supplement: Supplementary file 1 [file Image_1.tif]
